# Unravelling the enhanced vaccine immunity by heterologous KCONVAC/Ad5-nCoV COVID-19 vaccination

**DOI:** 10.1038/s41392-022-01079-8

**Published:** 2022-07-04

**Authors:** Weijun Zhao, Huajun Zhao, Baoying Huang, Tongyi Zhao, Limei Wang, Jian Zhang, Yong Yang, Xinying Tang, Wenjie Tan, Ang Lin

**Affiliations:** 1 Vaccine Center, School of Basic Medicine and Clinical Pharmacy; Center for New Drug Safety Evaluation and Research, China Pharmaceutical University, Nanjing, China; 2 Institute of Immunopharmaceutical Sciences, School of Pharmaceutical Sciences; Advanced Medical Research Institute, Shandong University, Jinan, China; 3 NHC Ley Laboratory of Biosafety, National Institute for Viral Disease Control and Prevention, Chinese Center for Disease Control and Prevention, Beijing, China

**Keywords:** Preclinical research, Vaccines

**Dear Editor**,

Growing evidence has indicated that heterologous COVID-19 vaccination could generate higher antibody (Ab) and cell-mediated immune (CMI) responses than homologous vaccination regimen.^1–3^ However, fundamental understanding of immunological mechanisms dictating the enhanced vaccine immunity is still lacking. To gain mechanistic insights, we comprehensively profiled the immune responses generated by homologous or heterologous booster vaccination in mice (Fig. 1a).

**Fig. 1:**
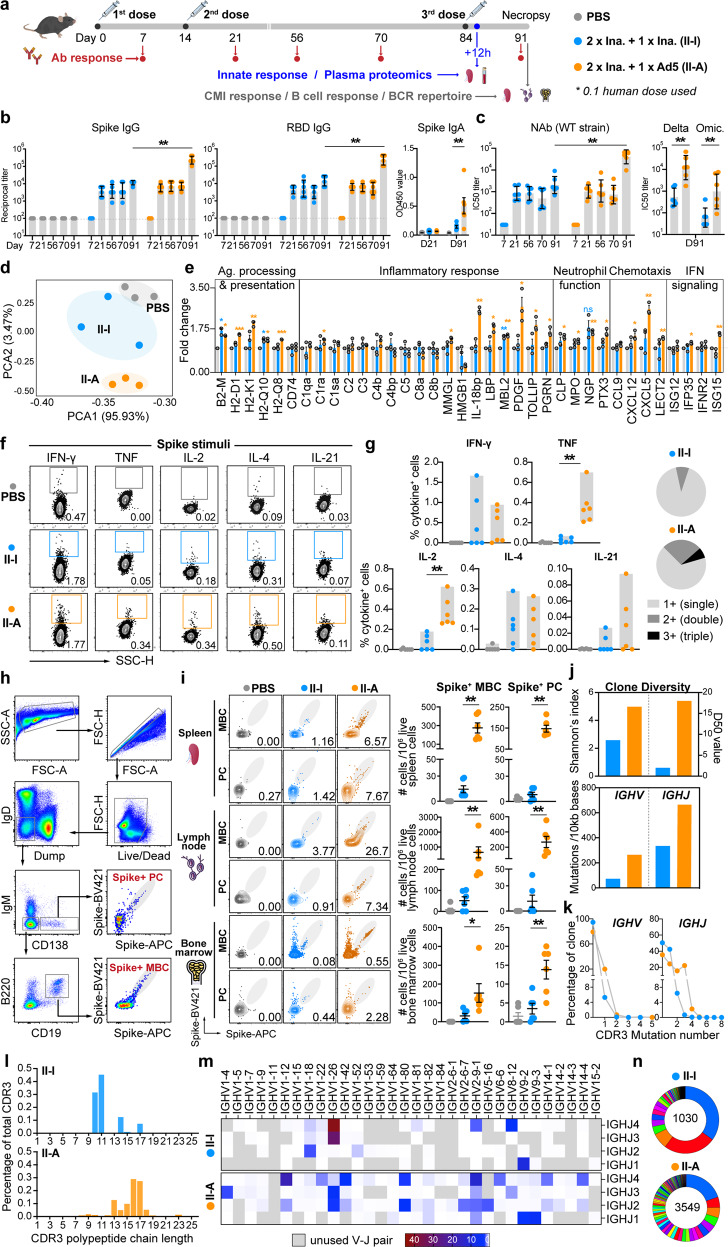
In-depth profiling of vaccine responses in mice receiving two-dose inactivated vaccines followed by homologous booster or heterologous booster with Ad5-nCoV. **a** Overview of study design. **b** Endpoint titers of Spike- and RBD-specific IgG are shown. Spike-specific IgA titer is depicted as OD450 value (*n* = 6). **c** Neutralizing Abs against SARS-CoV-2 wide-type strain, Delta and Omicron variants were measured using pseudotyped virus neutralizing test (*n* = 6). **d** PCA analysis of proteome in plasma collected 12 h following booster. **e** Quantification of indicated proteins in each group. Data is shown as fold change normalized to the PBS group (*n* = 3). Asterisks indicate the statistical difference compared to PBS group. **f** Splenocytes were stimulated with Spike protein (5 μg/ml) for 8 hours in the presence of Brefeldin A (*n* = 6). Frequencies of IFN-γ, TNF, IL-2, IL-4 or IL-21-secreting T cells were evaluated by flow cytometry. Representative data is shown. **g** Quantification of cytokine-producing memory CD4^+^ T cells in vaccinated mice (*n* = 6). Negative control (PBS) values were subtracted. The ability of T cells to produce single (1+), double (2+) or triple (3+) types of Th1-cytokines (IFN-γ, TNF, IL-2) was analyzed using SPICE software. **h** Representative gating strategy of Spike^+^ class-switched MBC and PC in spleen. **i** Frequencies of Spike^+^ class-switched MBCs and PCs in the indicated lymphoid organs were assessed (*n* = 6). **j** Shannon’s entropy index and D50 value indicating BCR clone diversity are shown. Somatic hypermutation (SHM) rate in the *IGHV* and *IGHJ* genes is shown. **k** Percentage of *IGHV* or *IGHJ* gene segments with mutated or unmutated CDR3 out of total reads. **l** Percentage and distribution of CDR3 with different length. **m** Frequency of usage of *IGHV-J* gene pair is shown. **n** Pie charts show the distribution of top 100 dominant BCR clones. The number inside the circle indicates the number of total sequences analyzed. *p ≤ 0.05, **p ≤ 0.01, ***p ≤ 0.001

C57BL/6 mice were immunized intramuscularly with two doses of inactivated vaccines (KCONVAC, Shenzhen Kangtai) at a 2-week interval, followed by either a homologous booster or a heterologous booster with Ad5-nCoV at week 12. Two doses of KCONVAC induced robust levels of Spike- and RBD-specific IgG, which reached plateau 1 week after 2nd dose (Fig. 1b). While a 3rd dose further stimulated the IgG response irrespective of vaccine type, the heterologous booster induced a significantly higher IgG titer than the homologous control. 7 days after booster, geometric mean titers (GMTs) of anti-Spike IgG in homologous and heterologous vaccination groups were 11,404 and 229,880, respectively. Interestingly, booster with Ad5-nCoV elicited a moderate anti-Spike IgA response (Fig. 1b), which offered additional advantage for heterologous vaccination strategy.^4^ Level of neutralizing Abs against SARS-CoV-2 wide-type strain was dramatically promoted upon heterologous booster and showed a 20-fold increase relative to that induced by homologous booster (Fig. 1c). Importantly, heterologous booster elicited a strikingly higher level of NAbs against the Delta (B.1.617.2) and Omicron (B.1.1.529) variants, albeit at a much lower level than that against the wide-type strain.

Early innate immune activation shapes the adaptive vaccine immunity. We therefore evaluated the innate responses 12 h after booster. Upon heterologous booster, a dramatic upregulation of CD86 on cDC1 from spleen and cDC2 from blood and spleen was observed, which was not seen in homologous booster group (Supplementary Fig. S1). Such response was likely attributed to the self-adjuvant effect of the vehicle since multiple signaling pathways involved in innate activation could be triggered by adenoviral vector itself. Mass spectrometry-based proteomics was employed to profile the global proteome. Principal component analysis of all quantified proteins revealed a distinct distribution among the three groups (Fig. 1d). Differentially expressed protein analysis revealed that heterologous booster significantly altered the plasma protein landscape, with 119 and 31 proteins showing at least 2-fold increased or decreased levels, respectively, when compared to PBS-treated mice. In contrast, levels of plasma proteins in mice receiving homologous booster largely resembled that in PBS-treated mice (Supplementary Fig. S2). Further analysis indicated that proteins with significant changes upon heterologous booster were highly involved in inflammatory responses, antigen uptake/processing and type I interferon response. Many of them showed a clear increase in mice receiving heterologous booster (Fig. 1e). Interestingly, both homologous and heterologous booster induced strong neutrophil activation, indicated by the release of granules such as myeloperoxidase, cathelicidin, neutrophilic granule protein and pentraxin-related protein 3. Splenic neutrophils were also activated, represented by the increased expression of CD40 and CD86 (Supplementary Fig. S3). In addition, booster with Ad5-nCoV increased plasma level of CXCL15, which is chemotactic for neutrophils and highly enriched in pulmonary compartment. Yet, neutrophil activation by COVID-19 vaccination has rarely been reported. Whether the activation of neutrophils is associated with the generation of vaccine responses awaits further investigation.

Th1-type cellular immunity is critical for the control of SARS-CoV-2 infection.^5^ Typically, inactivated vaccines adjuvanted with aluminum are more prone to elicit a Th2-type immunity. As was shown in our study, three-dose KCONVAC stimulated a robust level of IL-4-secreting CD4^+^ T cells (Fig. 1f, g). Only three out of six animals mounted specific CD4^+^ T cells producing IFN-γ or IL-2. While upon heterologous booster, frequencies of TNF- or IL-2-secreting CD4^+^ T cells were significantly elevated, and IL-4-secreting T cells were at a comparable level as that induced by homologous booster. A higher proportion of Spike-specific CD4^+^ T cells produced more than two types of Th1-type cytokines, indicating T cell polyfunctionality (Fig. 1g). IL-21-secreting CD4^+^ T cells were modestly induced, which suggested generation of T follicular helper cell response. These demonstrated that Ad5-nCoV booster following two-dose KCONVAC elicited a well-balanced Th1/Th2 vaccine response.

The enhanced Ab response by heterologous booster may be attributed to a robust germinal center (GC) reaction. To address this, spleen, lymph node (LN) draining injection site and bone marrow were next analyzed (Fig. 1h, i). Heterologous booster induced a strikingly higher level of class-switched Spike^+^ MBCs and Spike^+^ PCs in all three lymphoid organs (Fig. 1i). Somatic hypermutation (SHM) is a critical process during GC reaction that determines Ab affinity maturation and clone diversity. To assess this, CD19^+^IgD^-^IgM^-^Spike^+^ MBCs from spleen were sorted and subjected to BCR sequencing. Due to limited cell yield, sorted MBCs in each group were pooled together for analysis. Heterologous booster generated more diverse BCR clones, which was likely driven by the higher mutation rates of *IGHV* and *IGHJ* genes (Fig. 1j). The CDR3 showed higher mutation numbers in both *V* and *J* gene segments, accompanied by an increased length (Fig. 1k, l). The pair usage of *IGHV-IGHJ* gene segments was also assessed. In the homologous booster group, only limited numbers of heavy chain *V* and *J* genes were used, with *IGHV1-26-IGHJ3 and IGHV1-26-IGHJ4* pairs being the most frequently used. In contrast, BCR generated by heterologous booster showed a highly diverse and broad *V-J* gene usage, as well as much fewer unused pairs, of which *IGHV1-12-IGHJ4, IGHV2-9-1-IGHJ4, IGHV9-3-IGHJ1* were the three most abundantly used (Fig. 1m). The top 100 dominant BCR clones in each vaccine group were also identified and a marked increase in BCR clonality was observed in mice receiving heterologous booster (Fig. 1n). The Ig kappa (κ) and lambda (λ) light chains were also sequenced. For Ig λ repertoire, *IGLV1-IGLJ1* represented the most frequently used pair accounting for 98.4% and 99.1% of total *V-J* usages in homologous and heterologous booster group, respectively. While for Ig κ repertoire, heterologous booster generated a much broader *V-J* gene usage with *IGKV6-15-IGKJ1* being the most frequently used. In contrast, the BCR repertoire elicited by homologous booster predominantly used *IGKV3-1-IGKJ1* pair (Supplementary Fig. S4). These data together indicated that heterologous booster with Ad5-nCoV was more efficient at eliciting diverse Spike-specific Ab clones with a higher SHM rate, which was associated with the enhanced anti-viral Ab response.

Clinical evidence has indicated that heterologous COVID-19 vaccine booster was more advantageous than homologous booster, in terms of not only the induction of higher Ab responses but also more efficient protection. Using mouse model, we have comprehensively investigated on many of the aspects involved in the initiation, generation and maintenance of vaccine responses upon homologous or heterologous booster, which provided mechanistic explanations on some of the key questions regarding the enhanced vaccine immunity by heterologous booster. One limitation of our study is that the association between innate activation and generation of high-quality Ab response and GC reaction by heterologous booster was not dissected, which was mainly due to the animal model used and technical limitations. However, manipulating innate compartment via using optimal adjuvants has been widely believed to be a promising strategy that can be deployed for the next-generation COVID-19 vaccine development. A primary two-dose inactivated vaccine followed by adenoviral vectored vaccine booster may therefore hold prospect of tackling the challenges posed by continuously emerging SARS-CoV-2 variants and rapid waning immunity.

## Supplementary information

Supplemental information

supplemental fig 2

supplemental fig 1

supplemental fig 3

supplemental fig 4

## Data Availability

All data are available upon reasonable request to the corresponding author.

## References

[1] Atmar RL (2022). Homologous and Heterologous Covid-19 Booster Vaccinations. N. Engl. J. Med..

[2] Li J (2022). Heterologous AD5-nCOV plus CoronaVac versus homologous CoronaVac vaccination: a randomized phase 4 trial. Nat. Med..

[3] WHO. Interim recommendations for heterologous COVID-19 vaccine schedules. World Health Organization. (2021).

[4] Sheikh-Mohamed, S. et al. Systemic and mucosal IgA responses are variably induced in response to SARS-CoV-2 mRNA vaccination and are associated with protection against subsequent infection. *Mucosal Immunol* (2022). 10.1038/s41385-022-00511-0 PMC9037584 35468942

[5] Tan AT (2021). Early induction of functional SARS-CoV-2-specific T cells associates with rapid viral clearance and mild disease in COVID-19 patients. Cell Rep..

