# Supplementary material for: Unravelling the enhanced vaccine immunity by heterologous KCONVAC/Ad5-nCoV COVID-19 vaccination
Source: Signal Transduct Target Ther. 2022 Jul 4;7:210. doi: 10.1038/s41392-022-01079-8 (PMC9251036; doi:10.1038/s41392-022-01079-8)
Supplement: Supplementary file 1 — Supplemental information [file 41392_2022_1079_MOESM1_ESM.docx]

**Supplementary Material for**

**Unravelling the enhanced vaccine immunity by heterologous KCONVAC/Ad5-nCoV COVID-19 vaccination**

Weijun Zhao^1*^, Huajun Zhao^2*^, Baoying Huang^3*^, Tongyi Zhao^1*^, Limei Wang^2^, Jian Zhang^2^, Yong Yang^1^, Xinying Tang^1🖂^, Wenjie Tan^3🖂^, Ang Lin^1🖂^

**Affiliations:**

1. Vaccine Center, School of Basic Medicine and Clinical Pharmacy; Center for New Drug Safety Evaluation and Research; China Pharmaceutical University, Nanjing, China
2. Institute of Immunopharmaceutical Sciences, School of Pharmaceutical Sciences; Advanced Medical Research Institute; Shandong University, Jinan, China
3. NHC Ley Laboratory of Biosafety, National Institute for Viral Disease Control and Prevention, Chinese Center for Disease Control and Prevention, Beijing, China

*Authors contributed equally

**Correspondence to:**

Ang Lin, anglin@cpu.edu.cn; Wenjie Tan, tanwj@ivdc.chinacdc.cn; Xinying Tang, xinyingtang@cpu.edu.cn;

**Lead contact:**

Ang Lin, anglin@cpu.edu.cn

**This PDF file includes:**

Materials and Methods

Supplemental Figure 1, 2, 3, 4

**Materials and Methods**

**Immunization and sample collection**

C57BL/6 mice (male, 6-week old) were obtained from Beijing HFK Bioscience Co. Ltd. (Beijing, China) and randomly allocated to different vaccine groups. Inactivated COVID-19 vaccine (KCONVAC) and Ad5-nCoV5 vaccine were gifted from Shenzhen Kangtai Biological Products Co. Ltd and CanSino Biologics Inc, respectively. For immunizing mice, 0.1 human dose of the two vaccines was used. Mice (n=9 each group) were i.m. immunized with two doses of KCONVAC (10U) at day 0 and 14, followed by a 3^rd^ dose with KCONVAC or Ad5-nCoV5 (5×10^9^ VP) at week 12. Mice (n=9) received PBS injection were used as control. Sera samples were collected longitudinally on day 7, 21, 56, 70 and 91. At day 7 post the 3^rd^ immunization, mice (n=6 each group) were necropsied and spleen, lymph node and bone marrow were processed to obtain single cell suspension as previously described^1^. All animal experiments were performed in accordance with the Guidelines for the Care and Use of Laboratory Animals and the Ethical Committee of Shandong University and the protocols were approved by the Institutional Animal Care and Use Committee (approval number: 20023).

**Measurement of antigen-specific binding Ab**

For the measurement of binding IgG, Spike and RBD proteins (Sino Biological) were coated into 96-well plates (Greiner Bio-One) at a concentration of 100ng/well and incubated overnight at 4℃. The plates were then washed with PBS containing 0.05% Tween-20 (PBST) and subsequently blocked with 2% bovine serum albumin (BSA) for 2 h at 30℃. Following this, sera serially diluted in PBST containing 0.2% BSA were added and incubated for 2h at 30℃. Binding IgG was determined using HRP-conjugated goat-anti-mouse IgG Ab (1:50,000, Abcam) for 1 hour at 30℃. TMB substrate was used for development, and the absorbance was read at 450 nm wavelength. Endpoint titer was calculated as the dilution factor that emitted an optical density (OD) value above 2.1×background. For the measurement of binding IgA, sera samples were 1:100 diluted before added into plates coated with Spike protein. HRP-conjugated goat-anti-mouse IgA Ab (1:10,000, Southern Biotech) was used for analysis.

**Measurement of neutralizing Ab (NAb)**

Levels of NAbs against original Wuhan-1 strain, Delta (B.1.617.2) and Omicron (B.1.1.529) variants were measured by pseudotyped virus neutralization test (pVNT). pVNT assay was performed with commercially available pseudotyped virus following the manufacturer’s instructions (Beijing SanYao Science and Technology Development Co, China). In brief, serially diluted heat-inactivated sera were mixed with 50 μL of pseudoviruses (2×10^4^ TCID_50_/mL) and incubated at 37 °C for 1 hour. Vero cells were then digested and added to each well (2×10^4^/100 μl). After incubation at 37°C for 24 hours, cells were lysed and the luminescence was measured using BriteLite Plus Luciferase reagent (PerkinElmer, USA). IC50 value was calculated as the serum dilution at which the luminescence was reduced by 50% compared with virus control wells.

**Plasma proteomics analysis**

Plasma samples (n=3 each group) were collected at 12 hours after the 3^rd^ immunization for proteomics analysis. Proteins were extracted using RIPA buffer and High-Select™ Abundant Protein Depletion Resin Kit (Thermo) was used to remove the top 14 abundant proteins. After acetone precipitation, samples were subjected to reduction, alkylation, trypsin digestion, TMT (Tandem Mass Tag) labelling, desalting and fractionation sequentially. For each sample, 2 μg of total peptides were then subjected to nanoLC-MS/MS analysis using nanoUPLC EASYnLC1200 (Thermo) and Q Exactive HF-X Orbitrap mass spectrometer with a nano-electrospray ion source (Thermo). Data dependent acquisition (DDA) was performed in profile and positive mode with Orbitrap analyzer at a resolution of 120,000 (@200 m/z) and m/z range of 350-1600 for MS1. For MS2 acquisition, the resolution was set to 45k with a fixed mass of 110 m/z. The automatic gain control targets for MS1 and MS2 were set to 3E6 with maximal IT 30 ms and 1E5 with maximal IT96 ms, respectively. The top 20 most intense ions were fragmented by HCD with normalized collision energy (NCE) of 32%, and isolation window of 0.7 m/z. Peaks that were single charged and with charges exceeding 6 were excluded from the DDA procedure. Raw MS data were processed and analyzed using Proteome Discoverer software. MS spectra lists were searched against UniProt FASTA databases (uniprot-Mus+musculus-10090-2021-8. fasta). Peptide identification was performed with an initial precursor mass deviation of up to 10 ppm and a fragment mass deviation of 0.02 Da. Unique peptide and Razor peptide were used for protein quantification and total peptide amount for normalization. All the other parameters were reserved as default.

**Memory B cell and PC analysis**

Frequencies of Spike-specific MBCs and PCs were assessed by flow cytometry. For the preparation of Spike probes, biotinylated Spike proteins (Acro Biosystems) were conjugated with BV421- or APC-streptavidin (Biolegend) at a molar ratio of 4:1. Cells from spleen, bone marrow or lymph nodes were first incubated with Spike probes for 20 minutes, and then stained with Fixable Viability Dye eFluorTM 506 (eBioscience) for 5 minutes. After washing, cells were incubated with Fc Receptor blocking reagent (Miltenyi) and antibody cocktails for 20 minutes at 4℃ in dark. Flow cytometric analysis was carried out on BD FACSymphony A3 (BD Biosciences). Data were analyzed using FlowJo V.10.1 (Tree Star). Antibody cocktail contains the following fluorescently-labelled antibodies: BV605 anti-mouse IgD (clone: 11-26c.2a), APC-Cy7 anti-mouse IgM (clone: RMM-1), FITC anti-mouse CD3 (clone: 17A2), FITC anti-mouse F4/80 (clone: BM8), PE anti-mouse CD19 (clone: 6D5), Percp-Cy5.5 anti-mouse B220/CD45RA (clone: RA3-6B2), PE-Dazzle594 anti-mouse CD38 (clone: 90), BV786 anti-mouse CD138 (clone: 281-2).

**Evaluation of antigen-specific T cell response**

2 million splenocytes were seeded per well into 96-well U-bottom plates and incubated with Spike protein (5 μg/mL, Sino Biological) in the presence of brefeldin A (BFA, Biolegend) for 8 hours at 37 °C. Cytokine production of memory T cells was evaluated by surface and intracellular staining using Fixation/Permeabilization Solution Kit (BD Biosciences) as previously described^2^. Frequency of cytokine-producing T cells was determined by FACS analysis. Background cytokine staining was subtracted, as defined by staining in the samples incubated with medium alone. Polyfunctionality of antigen-specific T cells producing Th1-type cytokines (IFN-γ, TNF, IL-2) was analyzed using FlowJo V.10.1 (Tree Star) and SPICE software (v.6.0). Antibody cocktail contains the following fluorescently-labelled antibodies: FITC anti-mouse CD3 (clone: 17A2), BV785 anti-mouse CD4 (clone: GK1.5), Percp-Cy5.5 anti-mouse CD8 (clone:53-6.7), PE-Cy7 anti-mouse CD44 (clone: IM7), BV650 anti-mouse CD62L (clone: MEL-14), BV421 anti-mouse IFN-γ (clone: XMG1.2), PE-CF594 anti-mouse IL-2 (clone: JES6-5H4), APC anti-mouse TNF (clone: MP6-XT22), PE anti-mouse IL-21 (clone: mhalx21), BV605 anti-mouse IL-4 (clone: 11B11).

**Spike specific MBC sorting and BCR sequencing**

7 days after the 3^rd^ immunization (day 91), single-cell suspension of splenocytes was prepared as described above. CD19^+^ B cells were enriched using MojoSortTM mouse CD19 nanobeads (BioLegend) first and cell purity was above 90%. After this, cells were incubated with a mixture of Fc Receptor blocking reagent (Miltenyi), Spike probes and antibody cocktails (anti-mouse IgD and anti-mouse IgM) for 20 minutes. Spike^+^IgD^-^IgM^-^ MBCs were sorted using Moflo Astrios EQ (Beckman). Purity of sorted cells was confirmed by FACS as more than 95%. Total RNAs were extracted from MBCs using TRIzol (Invitrogen), followed by removal of DNA using DNaseI (Thermo). 2 μg RNA of each sample was used to prepare the BCR library using KC-Digital Stranded BCR-seq Library Prep Kit for Illumina® 150 (Seqhealth Technology Co., Ltd., Wuhan). Duplication bias was depleted using unique molecular identifier (UMI) of 8 random bases to tag the pre-amplified cDNA. After this, NovaSeq (Illumina) was used to sequence the library products with 250-500bp. Raw data was filtered using SOAPnuke (version 1.6.0) first, and low-quality reads were discarded. Consensus sequences were finally fished out and subjected to BCR-seq analysis. Reads were mapped to the international ImMunoGeneTics (IMGT) database using MiXCR software (v.3.0.3) to obtain V, D and J gene fragment and the CDR3 sequences. To quantify the BCR clones that were most frequently expanded, IGH sequences that used the same V(D)J alleles and had ≤1 mutation in CDR3 region were clustered. Top 100 dominant BCR clones were then identified. Raw BCR sequencing data for all mice are available on Sequence Read Archive (SRA) under BioProject: PRJNA833905.

**Statistical analysis**

Mann-Whitney U test was used to compare values between two different groups. Statistical difference among three different groups was analyzed by one-way analysis of variance (ANOVA). A *p* value less than 0.05 was considered statistically significant (*p < 0.05, **p < 0.01). Data were analyzed using GraphPad Prism v6.0.

**References**

1. Zhao, HJ. et al. Poly I: C-based rHBVvac therapeutic vaccine eliminates HBV via generation of HBV-specific CD8+ effector memory T cells. Gut. 11, 2032-2043, (2019)

2. Lin, A. et al. Live Live attenuated pertussis vaccine BPZE1 induces a broad antibody response in humans. J Clin Invest.130, 2332-2346. (2020)

**Supplemental Figure 1**

**
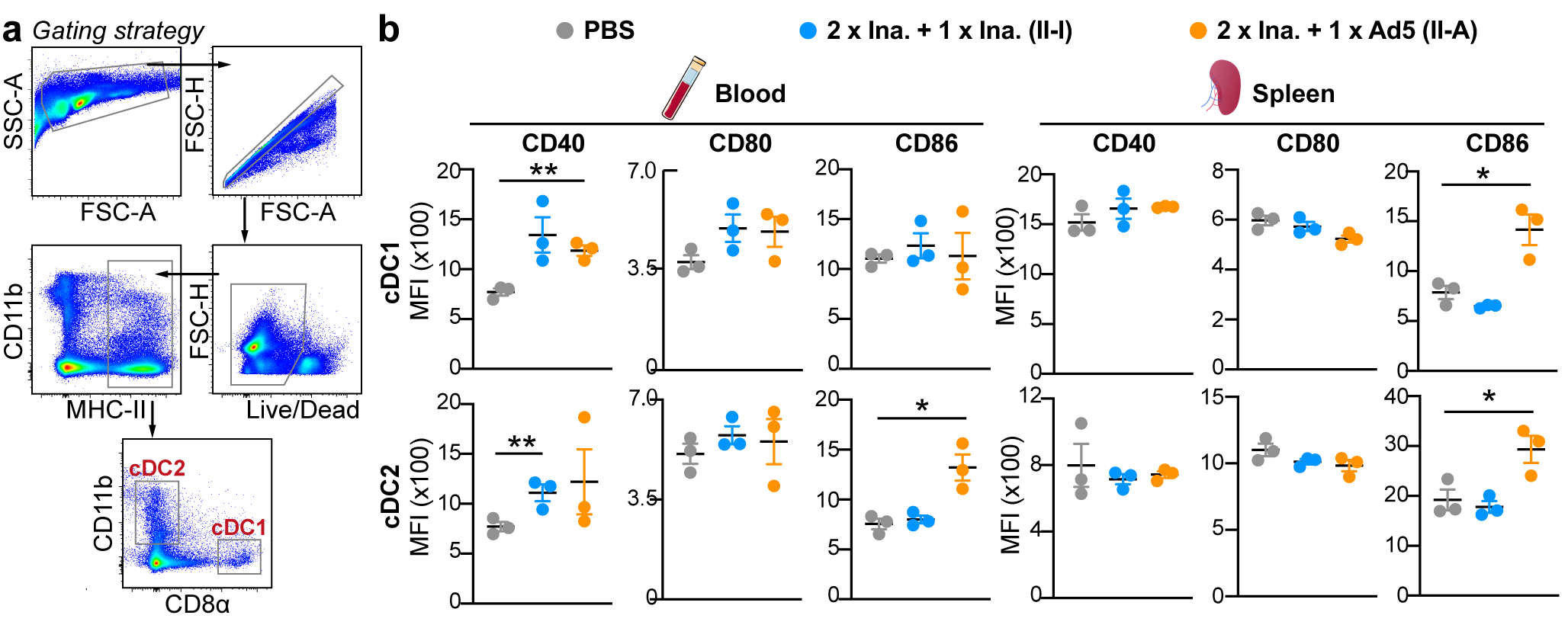
**

**Fig S1. Evaluation of innate activation following homologous or heterologous booster.**

**a**. Phenotypic identification of cDC1 and cDC2 cells in spleen. Gating strategy from one representative animal is shown. **b**. Surface expression of CD40, CD80 and CD86 on cDC1 and

cDC2. MFI values of the indicated markers are shown (n=3).

**Supplemental Figure 2**


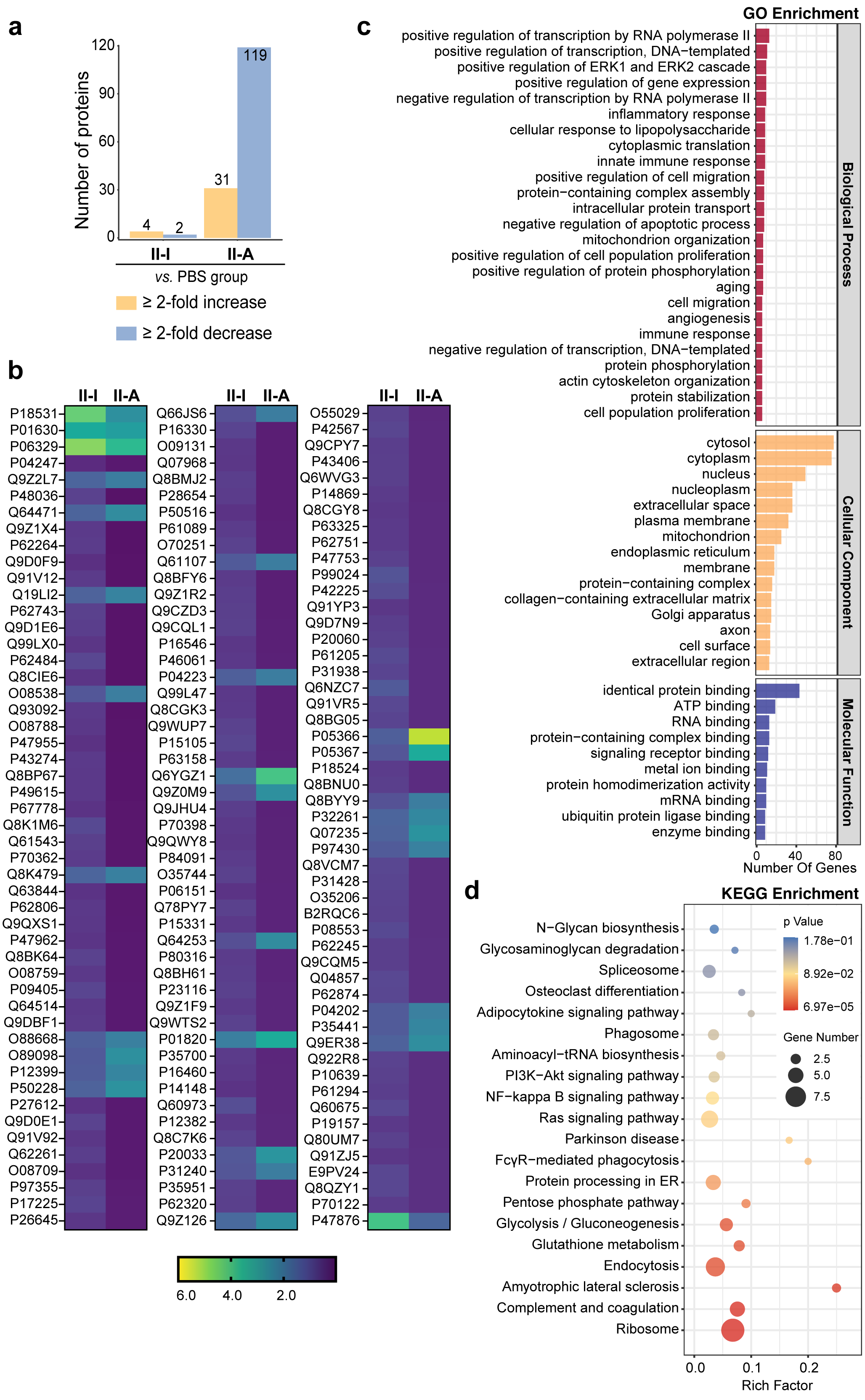


**Fig S2. Distinct plasma proteome signature of vaccinated mice following homologous or heterologous booster.**

**a-b**. Plasma proteins with ≥2-fold increased or decreased level compared to PBS-treated mice are shown. UniProt ID of each protein is shown. **c-d.** Enrichment analysis of KEGG pathways and GO terms in heterologous booster group versus PBS group.

**Supplemental Figure 3**


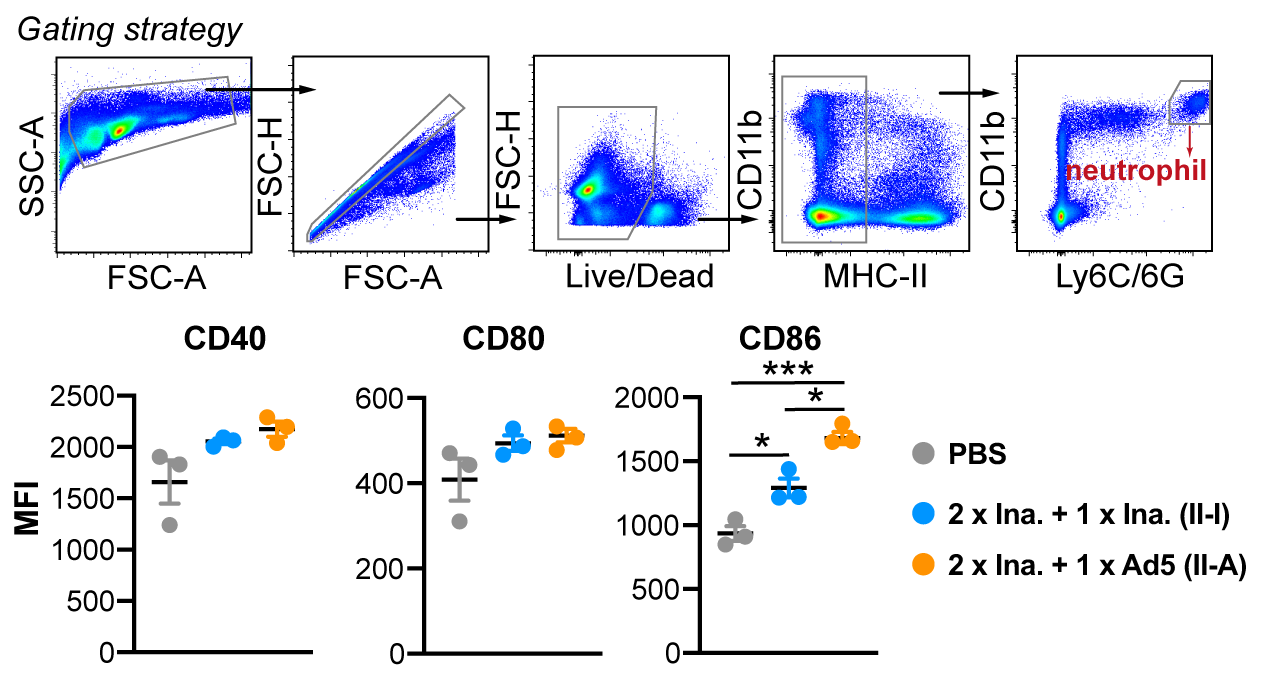


**Fig S3. Evaluation of neutrophil activation following homologous or heterologous booster.**

Phenotypic identification of neutrophils in spleen of mouse. Gating strategy from one representative animal is shown (upper panel). Surface expression of CD40, CD80 and CD86 on neutrophils was evaluated by flow cytometry. MFI values of the indicated markers are shown (n=3).

**Supplemental Figure 4**


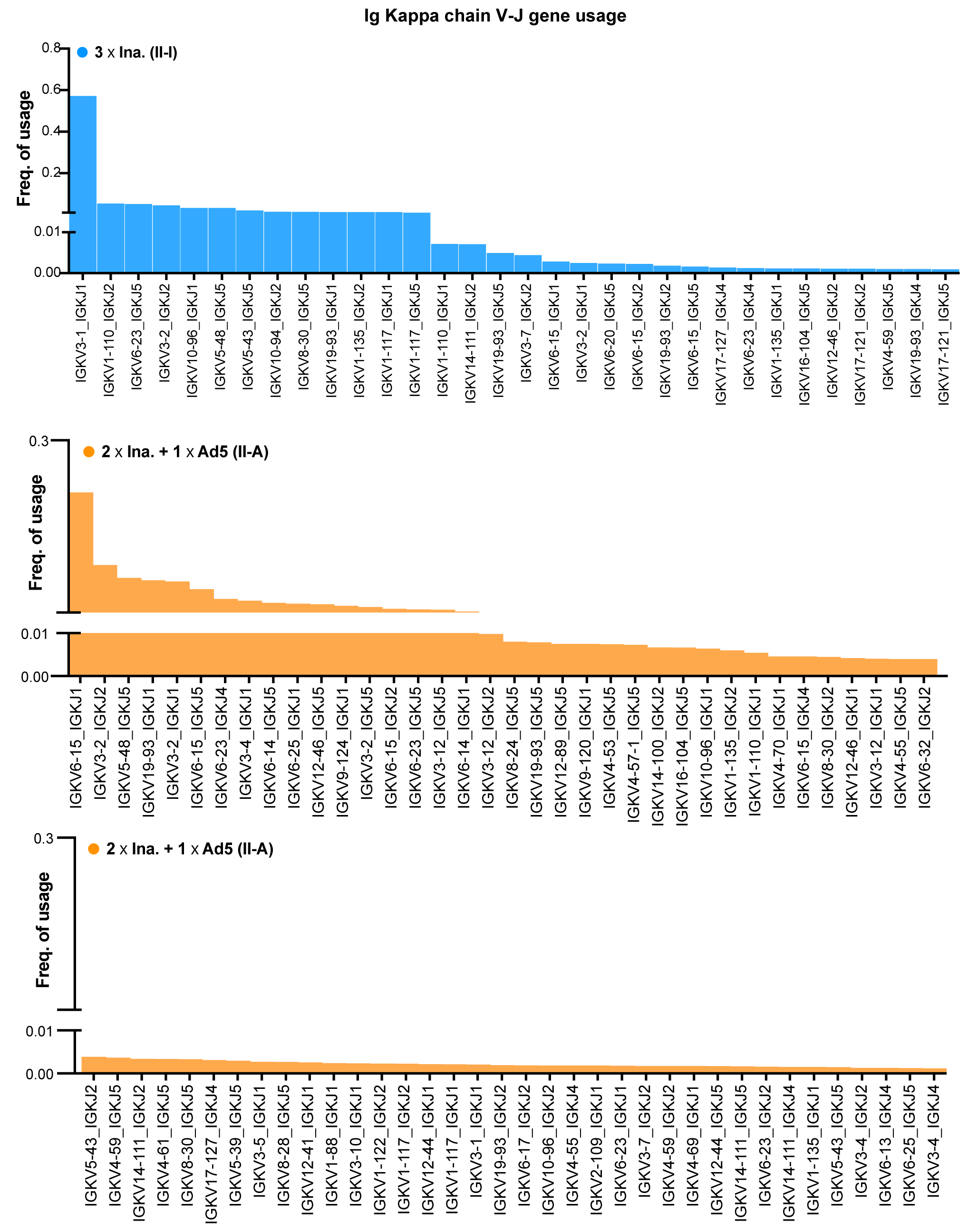


**Fig S4. *V-J* pair usage of Ig kappa light chain.** Frequency of *IGKV-J* gene pair usage is shown. Only the pairs used at a frequency above 0.001% are shown.
