# Supplementary figures and images for: Unravelling the enhanced vaccine immunity by heterologous KCONVAC/Ad5-nCoV COVID-19 vaccination
Source: Signal Transduct Target Ther. 2022 Jul 4;7:210. doi: 10.1038/s41392-022-01079-8 (PMC9251036; doi:10.1038/s41392-022-01079-8)

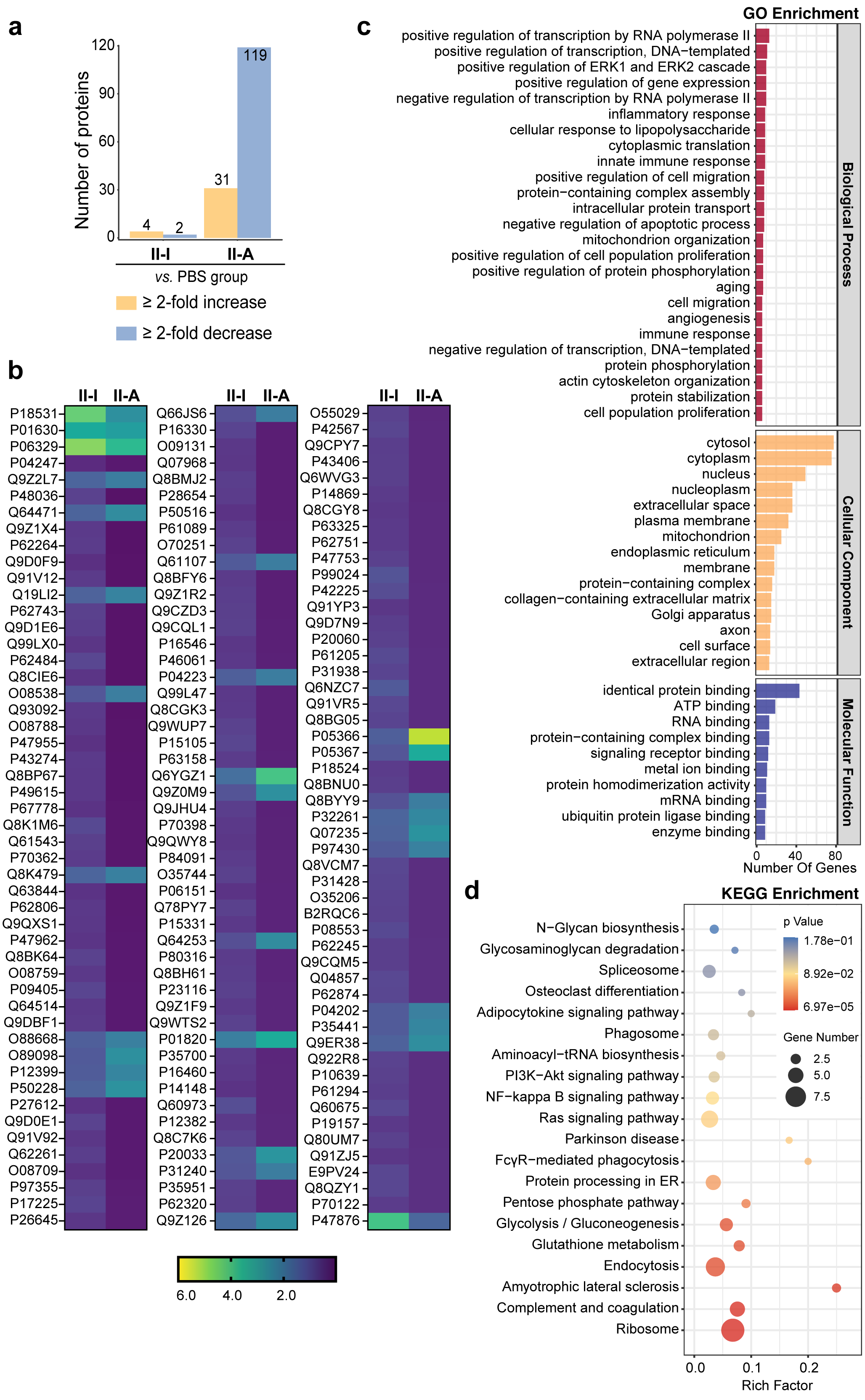

Supplement: Supplementary file 2 — supplemental fig 2 [file 41392_2022_1079_MOESM2_ESM.tif]

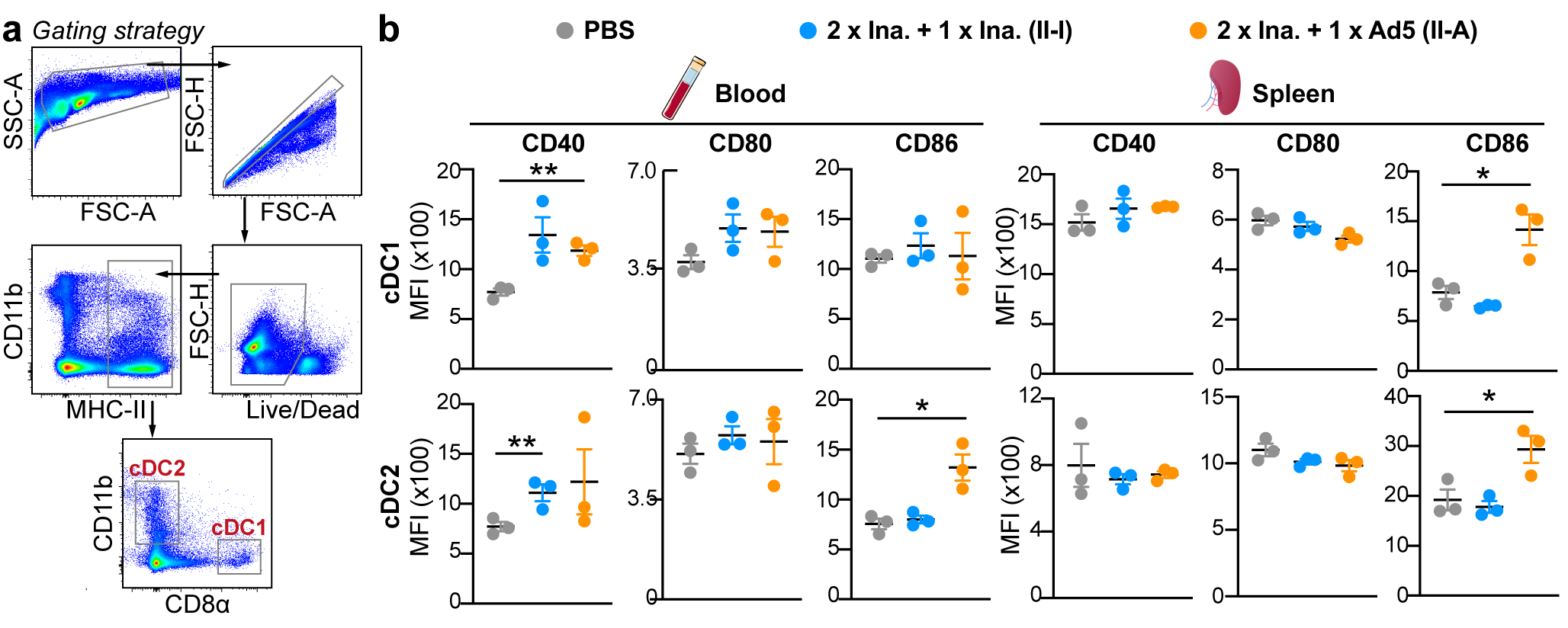

Supplement: Supplementary file 3 — supplemental fig 1 [file 41392_2022_1079_MOESM3_ESM.tif]

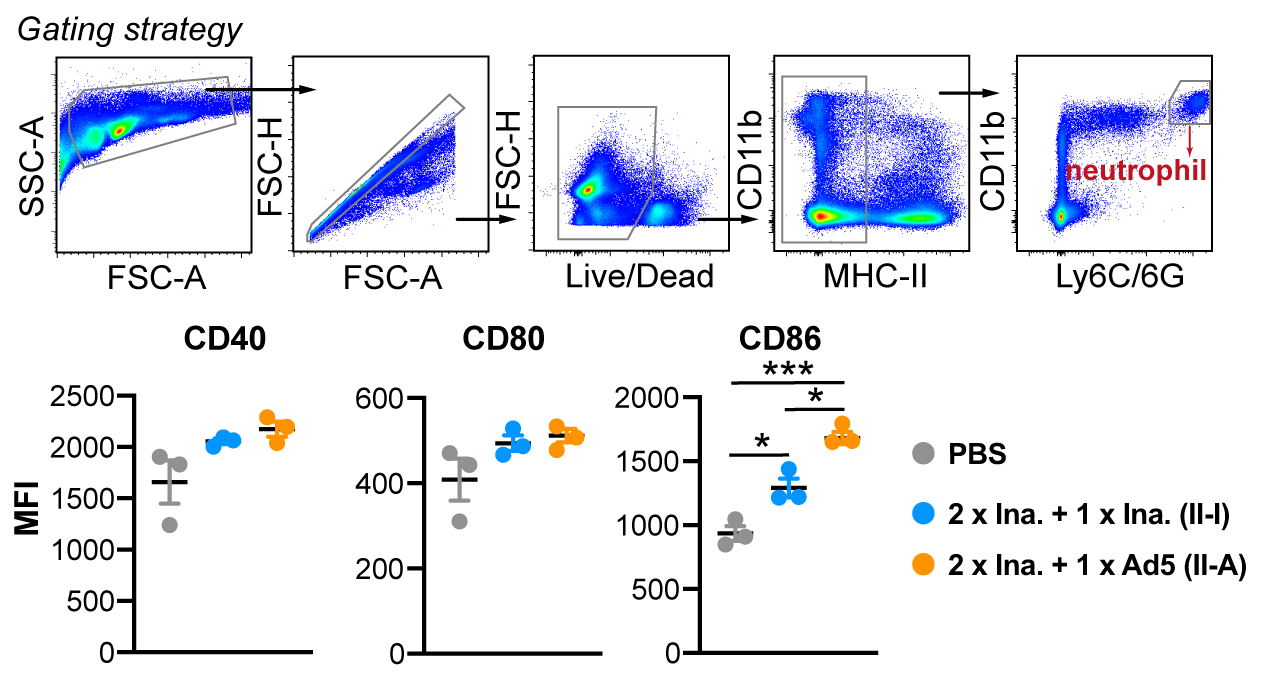

Supplement: Supplementary file 4 — supplemental fig 3 [file 41392_2022_1079_MOESM4_ESM.tif]

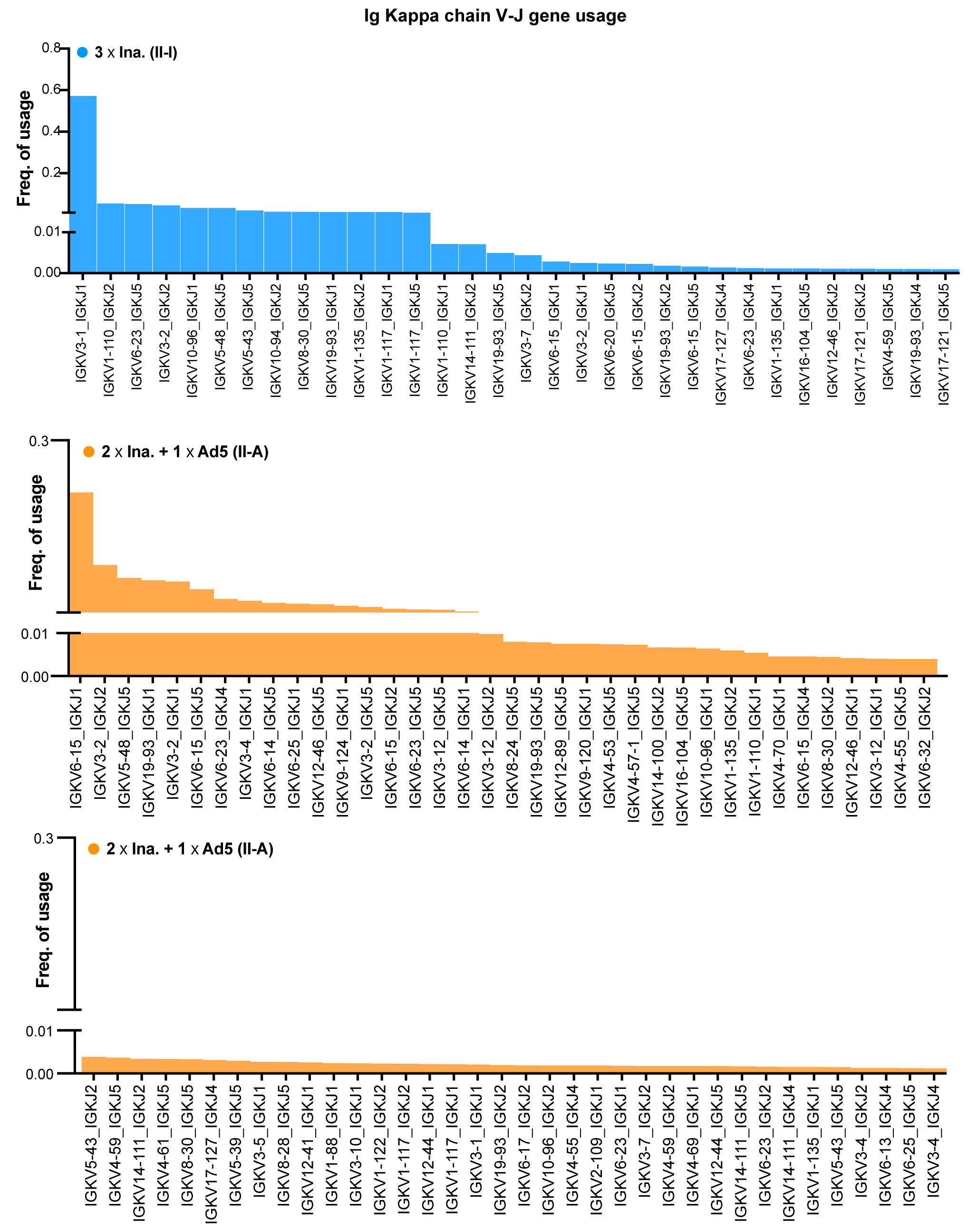

Supplement: Supplementary file 5 — supplemental fig 4 [file 41392_2022_1079_MOESM5_ESM.tif]
